# Antibody S22019F Selectively Recognises KIR2DS1 and Enables Analysis of KIR2DS1 ^+^ NK Cells and T Cells

**DOI:** 10.1111/tan.70748

**Published:** 2026-05-13

**Authors:** Eleni Bilev, Julia Meinecke, Jascha Wienberg, Caroline Boulouis, Michael Li, Takatoku Oida, Jakob Michaëlsson, Hans‐Gustaf Ljunggren, Petra Bacher, Carsten Wiethe, Quirin Hammer

**Affiliations:** ^1^ Center for Infectious Medicine, Department of Medicine Huddinge, Karolinska Institutet Karolinska University Hospital Huddinge Stockholm Sweden; ^2^ Institute of Medical Immunology Christian‐Albrechts‐University of Kiel and University Medical Center Schleswig‐Holstein Kiel Germany; ^3^ BioLegend Inc. San Diego California USA

**Keywords:** HLA‐C, killer cell immunoglobulin‐like receptors, KIR polymorphism, KIR2DS1, monoclonal antibodies, NK cells, T cells

## Abstract

Killer cell immunoglobulin‐like receptors (KIR) and their cognate HLA ligands regulate the functions of natural killer (NK) cells. However, extensive sequence homology within the KIR family limits the ability of monoclonal antibodies to selectively recognise individual receptors, and no KIR2DS1‐specific reagent is available to date. Here, we comprehensively delineate the binding profile of the monoclonal antibody S22019F. Using Ba/F3 cells, NK cell clones, and primary NK cells, we demonstrate that S22019F selectively binds KIR2DS1. Moreover, functional assays reveal that binding of S22019F to KIR2DS1 is preserved upon activation by its HLA‐C ligand. We further confirm that S22019F does not cross‐react to KIR2DL3*005 allotypes and we extend its application to the analysis of KIR2DS1^+^ T cells. Collectively, these findings underscore the value of S22019F as a reagent that selectively recognises KIR2DS1, enabling improved analyses of KIR2DS1‐bearing lymphocytes in fundamental and clinical research.

## 1 Introduction

Natural killer (NK) cells are cytotoxic innate lymphocytes that contribute to the first line of defence against virus infections and cancer [1]. A hallmark of NK cells is the expression of multiple germline‐encoded receptors that survey potential target cells for altered ligand expression and transmit activating or inhibitory signals, thereby regulating NK cell activity. Among this variety of receptors, killer cell immunoglobulin‐like receptors (KIR) constitute a highly polymorphic receptor family that specifically bind to groups of HLA‐A, ‐B, or ‐C allotypes [2], with varying degrees of peptide selectivity [3]. KIR molecules are composed of 2 or 3 Ig‐like extracellular domains (2D or 3D) and, depending on the length of their intracellular tail, transmit either inhibitory (long, L) or activating (short, S) signals to regulate NK cell activity. Interactions between inhibitory KIR such as KIR2DL1 or KIR2DL3 and their cognate HLA ligands mediate NK cell education and maintain tolerance to self [2, 4], whereas activating KIR including KIR2DS1 or KIR2DS2 drive NK cell responses against malignantly transformed and infected cells [5, 6, 7].

Extensive sequence homology within the KIR family limits the ability of monoclonal antibodies to selectively recognise individual receptors. For instance, no KIR2DS1‐specific reagent is currently available. The antibody EB6, which binds both KIR2DL1 and KIR2DS1 [8], is commonly used to study KIR2DS1‐expressing cells. Due to the dual‐specificity of EB6, identification of KIR2DS1^+^KIR2DL1^−^ NK cells requires a combinatorial approach, in which EB6 is used together with an antibody specific for KIR2DL1 such as REA284, #1432111, or HP‐DM1. Using this combination, KIR2DL1^+^ cells can be excluded from the KIR2DS1/KIR2DL1^+^ population to indirectly identify KIR2DS1^+^KIR2DL1^−^ cells. The absence of reagents exclusively recognising KIR2DS1 has impeded in‐depth studies of its expression patterns, regulation, and functional contributions.

Here, we comprehensively characterise the binding profile of the recently generated monoclonal antibody S22019F. Using Ba/F3 cells, NK cell clones, and primary NK cells, we demonstrate that S22019F selectively binds KIR2DS1. Moreover, functional assays reveal that binding of S22019F to KIR2DS1 is preserved following activation by its ligand HLA‐C*15. Finally, we confirm that S22019F does not cross‐react to KIR2DL3*005 allotypes and we extend its application to the analysis of KIR2DS1^+^ T cells, highlighting broad utility of the antibody.

Taken together, these findings underscore the value of S22019F as a reagent that selectively recognises KIR2DS1, enabling improved analyses of KIR2DS1‐bearing lymphocytes in fundamental and clinical research.

## 2 Methods and Materials

### 2.1 Cells and Cell Lines

Ba/F3 cells were maintained in complete RPMI‐1640 (RPMI‐1640 supplemented with 2 mM glutamine, 10% [V/V] foetal bovine serum [FBS], 100 U/mL penicillin, and 100 μg/mL streptomycin; all Gibco) supplemented with 10 ng/mL recombinant mouse IL‐3 (BioLegend). To engineer KIR expression, Ba/F3 cells were transduced with a retrovirus encoding GFP together with KIR2DL1*003 (NM_014218.3), KIR2DS1*002 (NM_014512.1), KIR2DL3*001 (NM_015868.3), KIR2DL3*005 (AF022048), or KIR2DS5*002 (NM_014513).

Wildtype and HLA‐C*15‐expressing 721.221 cells were a kind gift of Peter Parham (Stanford University) and maintained in complete RPMI‐1640 in the absence and presence of 1 mg/mL Geneticin (Gibco), respectively. Surface expression HLA‐C*15 was confirmed by incubation with 50 μg/mL recombinant KIR2DL1‐Fc chimera proteins (RnD), followed by detection with polyclonal goat anti‐human IgG Fc‐PE secondary antibody (ThermoFisher; Table S1).

Buffy coats from healthy volunteer blood donors were obtained through the Department of Clinical Immunology and Transfusion Medicine, Karolinska University Hospital, after obtaining informed consent and as approved by the Ethical Review Authority (DNR 2025‐01197‐01). PBMC were isolated with standard density gradient centrifugation, cryopreserved in FBS containing 10% (V/V) DMSO (Sigma Aldrich), and thawed in the presence of Benzonase (Merck) as described previously [9].

### 2.2 S22019F Antibody

The mouse IgG1 monoclonal antibody S22019F was generated by BioLegend using standard procedures. In brief, splenocytes from BALB/c mice immunised with KIR2DS1‐transduced Ba/F3 cells were fused with SP2/0 myeloma cells. Hybridoma cells were screened by flow cytometry and clone S22019F was selected due to high reactivity for KIR2DS1 and low reactivity for KIR2DL1, KIR2DL2, KIR2DL3, KIR2DL4, KIR2DL5, KIR2DS2, KIR2DS3, KIR2DS4, KIR2DS5, KIR3DL1, KIR3DL2, KIR3DL3, and KIR3DS1. S22019F is commercially available from BioLegend.

### 2.3 Flow Cytometry

Antibody staining and flow cytometric analyses were performed following established guidelines [10]. In brief, cell suspensions were incubated with combinations of fluorochrome‐conjugated antibodies (Table S1) at optimised concentrations in PBS for 20 min at RT. Dead cells were identified with Zombie Aqua or Zombie NIR Fixable Viability Kit (both BioLegend) and excluded from analyses. Samples were fixed using BD Cytofix/Cytoperm (BD Biosciences) and, if indicated, intracellular proteins were stained with fluorochrome‐conjugated antibodies in Perm/Wash buffer (BD Biosciences) overnight (15–18 h) at 4°C. KIR2DL1 (REA284) and KIR2DL1/2DS1 (EB6) were stained using a sequential approach, with REA284 used first and EB6 second, as we previously described [11]. Samples were acquired on an LSR Fortessa flow cytometer (BD Biosciences) and analysed with FlowJo v10.10.0 (BD Biosciences) and SPICE [12].

### 2.4 Generation of Clonal NK Cell Populations

To obtain NK cell populations with homogenous KIR expression, NK cells were magnetically isolated from PBMC using the NK cell isolation kit (Miltenyi) and subsequently index sorted as viable CD3^−^ CD56^dim^ KIR2DL1^−^KIR2DS1^−^, KIR2DL1^+^KIR2DS1^−^, or KIR2DL1^−^KIR2DS1^+^ cells on a Discover S8 sorter (BD) using EB6 in combination with REA284. Single NK cells were directly sorted into U‐bottom 96 well plates pre‐seeded with irradiated mixed feeder cells (1.5 × 10^4^ 721.221 cells expressing membrane‐bound IL‐21 and 3 × 10^4^ PBMC pooled from four healthy donors per well) in SCGM (CellGenix) supplemented with 10% (V/V) human serum (Sigma Aldrich), 100 U/mL penicillin, 100 μg/mL streptomycin (both Gibco), 20 ng/mL IL‐15 (RnD), 20 ng/mL IL‐7 (PeproTech), and 40 U/mL IL‐2 (Miltenyi). Single NK cells were expanded for 4–6 weeks, with weekly media exchange. After visual confirmation of cell growth, clonal populations were collected and stained for flow cytometric analyses.

### 2.5 Sorting and Reverse Transcription Quantitative PCR (RT‐qPCR)

To determine transcript expression, NK cells were magnetically isolated from PBMC using the NK cell isolation kit (Miltenyi) and subsequently sorted as viable CD3^−^ CD56^dim^ KIR2DL1^−^KIR2DS1^−^, KIR2DL1^+^KIR2DS1^−^, or KIR2DL1^−^KIR2DS1^+^ cells on a MA900 sorter (Sony), using either EB6 in combination with REA284 or S22019F in combination with REA284. Total RNA was isolated from sorted cells using the RNeasy Micro Plus Kit (Qiagen) and RT‐qPCR was performed as previously described [13]. In brief, RNA was reverse transcribed using the QuantiTect Reverse Transcription Kit (Qiagen) and qPCR was performed in triplicates with the QuantiTect SYBR Green PCR Kit (Qiagen) and predesigned QuantiTect primer assays (Hs_GAPDH_1_SG [QT00079247], Hs_KIR2DL1_1_SG [QT01003422], and Hs_KIR2DS1_1_SG [QT01003436]) in a QuantStudio 5 instrument (ThermoFisher). Data were analysed using ThermoFisher Connect (ThermoFisher) and are presented relative to *GAPDH* as an endogenous control.

### 2.6 Co‐Culture With Target Cells

To investigate functional responses, PBMC were rested in complete RPMI‐1640 supplemented with 1 ng/mL IL‐15 (RnD) overnight (15–18 h) and on the next day, 5 × 10^5^ PBMC were co‐cultured with 5 × 10^4^ 721.221–wt or 721.221–HLA‐C*15 target cells for 4 h in V‐bottom 96‐well plates at 37°C. Degranulation was detected by the addition of anti‐CD107a antibody (Table S1) at the start of the co‐culture and GolgiStop as well as GolgiPlug (containing monensin and brefeldin A, respectively, both BD Biosciences) were added after 1 h. After 4 h total co‐culture time, the cells were stained for surface markers (Table S1), fixed, permeabilised, and stained for intracellular IFN‐γ and TNF‐α (Table S1) as described above. NK cells were gated within PBMC as viable CD14^−^ CD19^−^ CD3^−^ CD56^dim^ cells and further sub‐gated into KIR2DL1^+^KIR2DS1^−^ and KIR2DL1^−^KIR2DS1^+^ populations as indicated in Figure 5B,D.

### 2.7 Detection of KIR2DL3*005

To identify the presence of E35‐containing alleles such as KIR2DL3*005, KIR2DL3*010, or KIR2DL3*036, we screened 317 healthy donors for unusual staining patterns between GL183 and EB6 [14, 15] and isolated DNA from PBMC of four candidate donors using the QIAamp DNA Micro Kit (Qiagen). PCR was performed with an internal control as reported previously [15, 16]. Q5 High‐Fidelity 2X Master Mix (NEB) was used with 10 μM KIR2DL3*005_fwd 5′‐CAG AAA ACC TTC CCT CCG, 10 μM *KIR2DL2/3*_rev 5′‐TGG GCC CTG CAG AGA A, 1 μM *HLA‐DRA_fwd* 5′‐GAG GTA ACT GTG CTC ACG AAC AGC, and 1 μM *HLA‐DRA_rev* 5′‐CAC GTT CTC TGT AGT CTC TGG G, and results were visualised on a 1% (m/V) agarose (Invitrogen) gel as indicated in Figure S3A.

### 2.8 Statistics

Statistical analyses were performed in Prism 10 (GraphPad Software) and statistical parameters (sample size, employed tests, and significance) are reported in the figures and figure legends, with *p* ≥ 0.05 considered not significant (NS) and *p < 0.05, **p < 0.01, ***p < 0.001, and ****p < 0.0001. Two groups of paired samples were analysed with paired *t*‐test and three or more groups of samples were analysed with one‐way ANOVA (unpaired) or repeated‐measures one‐way ANOVA (paired) followed by Šídák's multiple comparisons test.

## 3 Results

### 3.1 S22019F Preferentially Binds KIR2DS1 on Transduced Ba/F3 Cells

**FIGURE 1:**
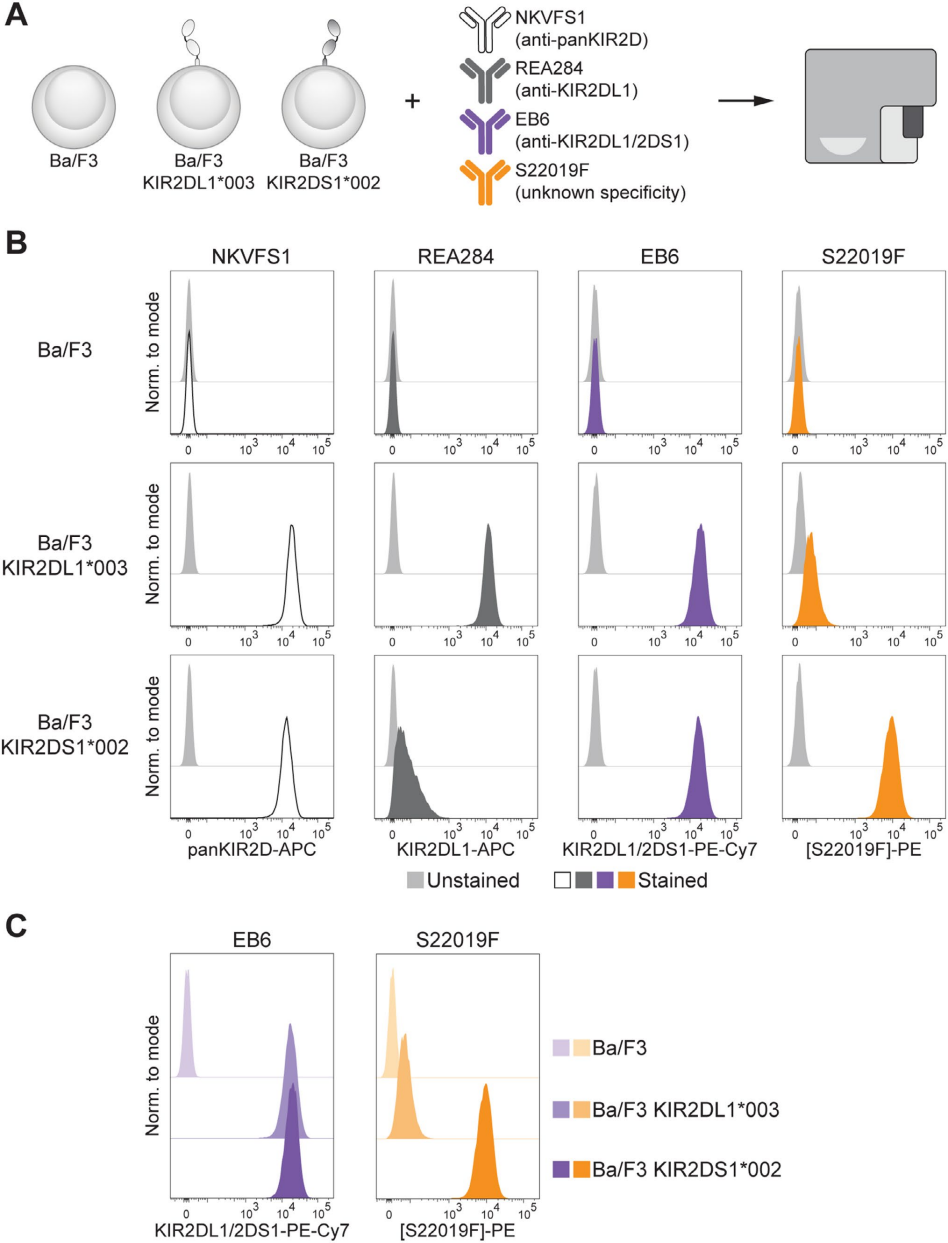
S22019F preferentially binds KIR2DS1 on transduced Ba/F3 cells. (A) Schematic overview of antibody binding assay. (B) Binding of anti‐panKIR2D NKVFS1, anti‐KIR2DL1 REA284, anti‐KIR2DL1/2DS1 EB6 and S22019F to unmodified, KIR2DL1*003‐transduced, and KIR2DS1*002‐transduced Ba/F3 cells. (C) Side‐by‐side comparison of EB6 and S22019F binding patterns to Ba/F3 cells. One representative of two experiments is displayed.

To assess whether the recently generated antibody S22019F specifically recognises KIR2DS1 and distinguishes between KIR2DL1 and KIR2DS1, we evaluated its binding profile by staining unmodified, KIR2DL1*003‐transduced, and KIR2DS1*002‐transduced Ba/F3 cells (Figure 1A). Successful transduction was confirmed by the anti‐panKIR2D antibody NKFVS1, and as expected, the anti‐KIR2DL1 antibody REA284 (antigen‐binding domain identical to #143211) preferentially bound KIR2DL1‐transduced Ba/F3 cells (Figure 1B). In line with its described dual specificity for both KIR2DL1 and KIR2DS1 [8], antibody EB6 labelled KIR2DL1‐transduced and KIR2DS1‐transduced Ba/F3 cells (Figure 1B). S22019F showed preferential binding to Ba/F3 cells transduced with KIR2DS1 (Figure 1B) and, consequently, S22019F clearly outperformed EB6 in a side‐by‐side comparison since it effectively distinguished Ba/F3 cells expressing KIR2DS1 from those expressing KIR2DL1 (Figure 1C).

These data indicate that S22019F preferentially binds KIR2DS1 on transduced Ba/F3 cells.

### 3.2 S22019F Selectively Binds KIR2DS1 on NK Cell Clones

**FIGURE 2:**
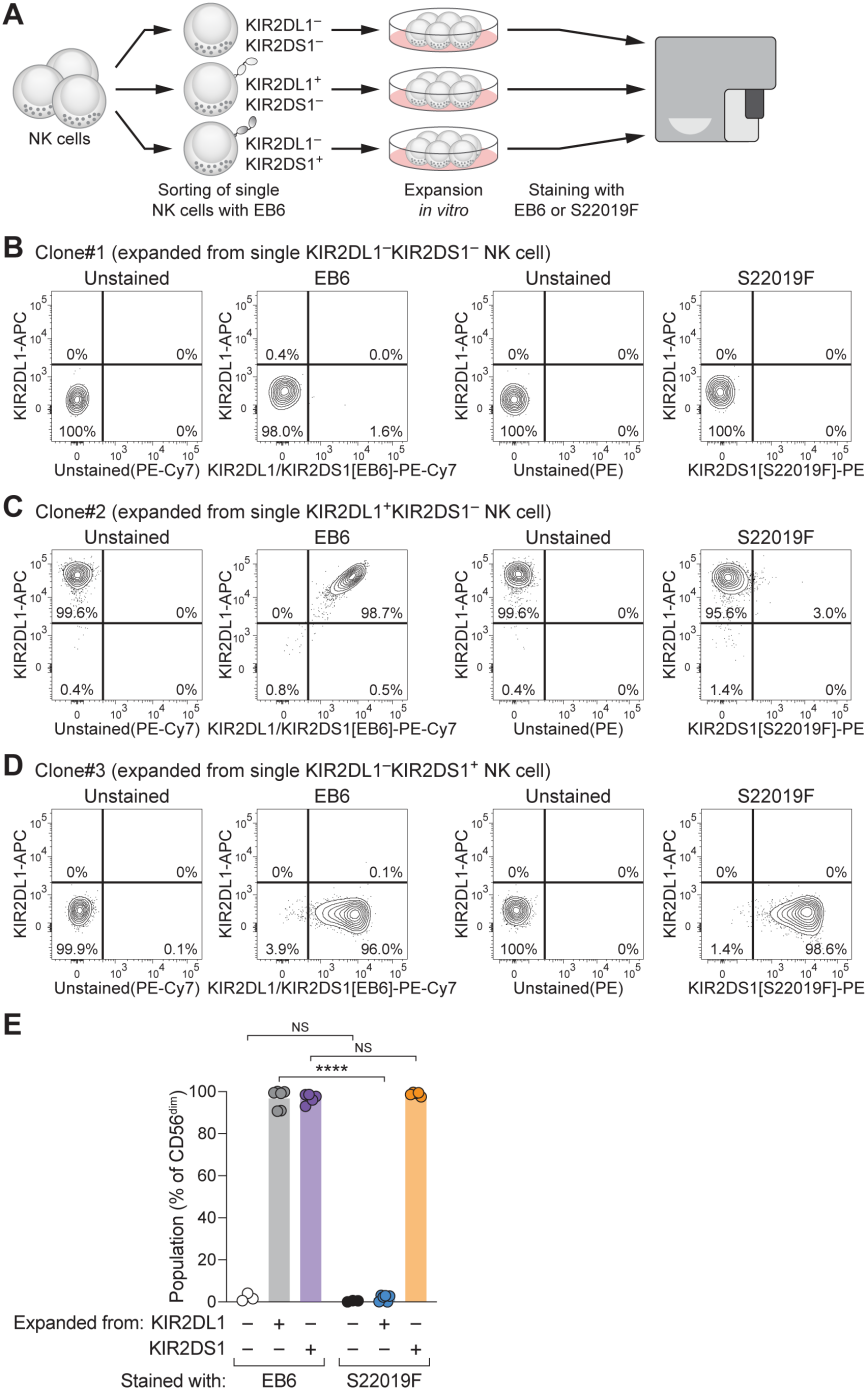
S22019F selectively binds KIR2DS1 on NK cell clones. (A) Schematic overview of NK cell clone expansion and re‐analysis. (B) Representative binding of anti‐KIR2DS1/2DL1 EB6 (left) and anti‐KIR2DS1 S22019F (right) in combination with REA284 to an NK cell clone expanded from a single KIR2DL1^−^KIR2DS1^−^ NK cell. (C) Representative binding as in (B) to an NK cell clone expanded from a single KIR2DL1^+^KIR2DS1^−^ NK cell. (D) Representative binding as in (B) to an NK cell clone expanded from a single KIR2DL1^−^KIR2DS1^+^ NK cell. (E) Summary of antibody binding to NK cell clones expanded from single cells with the indicated KIR profiles (*n* = 3 KIR2DL1^−^KIR2DS1^−^ clones, *n* = 6 KIR2DL1^+^KIR2DS1^−^ clones, and *n* = 5 KIR2DL1^−^KIR2DS1^+^ clones, bars indicate mean and each dot represents an individual clone; one‐way ANOVA with Šídák multiple comparison test). NS: Not significant, ****p <  0.0001.

Since S22019F preferentially bound to murine Ba/F3 cells engineered to express KIR2DS1, we next asked whether S22019F allows us to unequivocally identify human KIR2DS1^+^ NK cells. To obtain NK cells with homogenous KIR expression, we sorted single NK cells with defined single KIR2DL1 or KIR2DS1 profiles using EB6 in combination with REA284 and expanded them in vitro for re‐analysis of clonal populations with EB6 or S22019F (Figure 2A). NK cell clones expanded from KIR2DL1^−^ KIR2DS1^−^ cells served as negative controls and were not labelled by EB6 or S22019F (Figure 2B). In line with its dual specificity, EB6 bound NK cell clones expanded from KIR2DL1^+^ KIR2DS1^−^ cells as well as those expanded from KIR2DL1^−^ KIR2DS1^+^ cells (Figure 2C–E). In stark contrast, binding of S22019F to KIR2DL1^+^ KIR2DS1^−^ cells was virtually absent and S22019F efficiently marked all KIR2DL1^−^ KIR2DS1^+^ NK cell clones (Figure 2C–E).

Thus, these data show that S22019F selectively binds KIR2DS1 on NK cell clones.

### 3.3 S22019F Specifically Detects KIR2DS1 on Primary NK Cells

**FIGURE 3:**
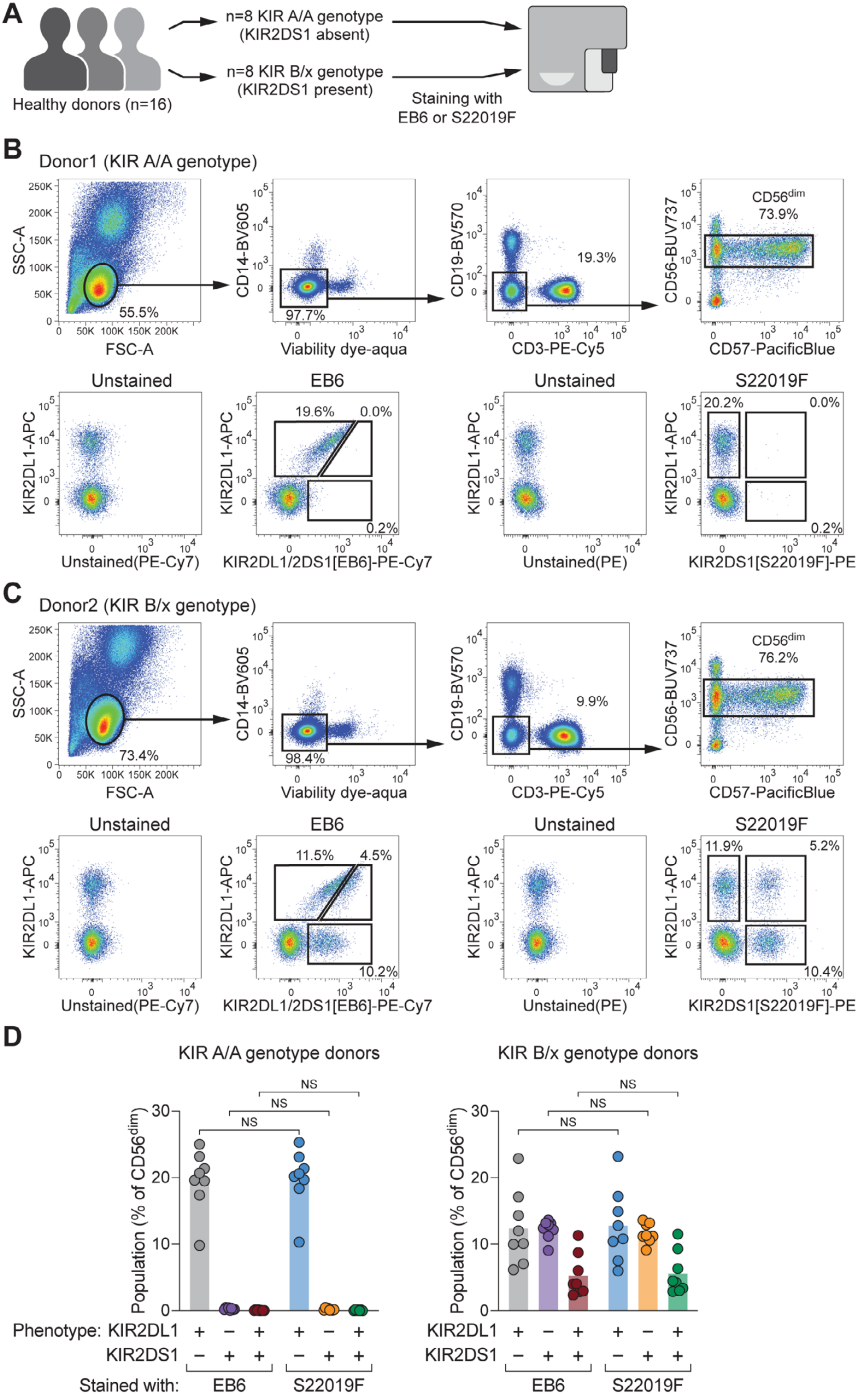
S22019F specifically detects KIR2DS1 on primary NK cells. (A) Schematic overview of flow cytometric analysis of PBMC from healthy donors with defined KIR genotypes. (B) Representative gating of viable CD14^−^ CD19^−^ CD3^−^ CD56^dim^ NK cells in a KIR A/A genotype donor (upper row) and staining patterns of EB6 (lower row, left) as well as S22019F (lower row, right) in combination with REA284. (C) Representative gating of viable CD14^−^ CD19^−^ CD3^−^ CD56^dim^ NK cells in a KIR B/x genotype donor (upper row) and staining patterns of EB6 (lower row, left) as well as S22019F (lower row, right) in combination with REA284. (D) Summary of frequencies of KIR2DL1^+^KIR2DS1^−^, KIR2DL1^−^KIR2DS1^+^, and KIR2DS1^+^KIR2DL1^+^ NK cell populations detected with EB6 or S22019F in KIR A/A (left) or B/x genotype (right) donors (*n* = 8 donors for each genotype, bars indicate mean and each dot represents an individual donor; repeated‐measures one‐way ANOVA with Šídák multiple comparison test). NS: Not significant.

Given that S22019F reliably distinguished between KIR2DS1 and KIR2DL1 on Ba/F3 cells and NK cell clones, we next evaluated its ability to detect KIR2DS1 on primary NK cells. For this, we incorporated S22019F into a multicolor flow cytometry panel and stained PBMC from 16 donors with known KIR genotypes (Figure 3A).

In genotype A/A donors, who lack the *KIR2DS1* gene, the combination of anti‐KIR2DL1/2DS1 antibody EB6 with anti‐KIR2DL1 (REA284) produced the expected diagonal staining pattern of KIR2DL1^+^ cells within viable CD14^−^ CD19^−^ CD3^−^ CD56^dim^ NK cells (Figure 3B). In contrast, S22019F showed no binding in these donors (Figure 3B), corroborating its advantageous specificity relative to EB6, and further indicating that it does not cross‐react to other KIR present in these donors.

In donors carrying the *KIR2DS1* gene and thus containing at least one B haplotype (B/x genotype), S22019F identified a discrete population of KIR2DS1^+^ cells. When combined with anti‐KIR2DL1 (REA284), S22019F precisely resolved KIR2DS1^−^KIR2DL1^+^, KIR2DS1^+^KIR2DL1^−^, and KIR2DS1^+^KIR2DL1^+^ NK cells, and as such provided a much clearer staining pattern compared to EB6 (Figure 3C).

To further assess the binding profile of S22019F, we analysed genotype B/x donors with a comprehensive panel covering KIR2DL1 (REA284), KIR2DL1/2DS1 (EB6), KIR2DL3 (REA147), KIR2DL2/2DL3/2DS2 (GL183), KIR3DL1 (DX9), and KIR2DS4 (REA860) (Figure S1A,B). Boolean gating revealed that the only KIR^+^ population that significantly positively correlated with the KIR2DS1^+^ cells detected by S22019F was the KIR2DS1^+^ population detected by using EB6 combined with anti‐KIR2DL1 (REA284) (Figure S1C), providing an unbiased validation that S22019F detects solely KIR2DS1 on NK cells.

Importantly, S22019F resulted in equivalent frequencies of KIR2DL1^+^KIR2DS1^−^, KIR2DL1^−^KIR2DS1^+^, and KIR2DL1^+^KIR2DS1^+^ cells compared to EB6 across all 16 tested donors (Figure 3D), emphasising concordant binding patterns.

Altogether, these data demonstrate that S22019F specifically detects KIR2DS1 on primary NK cells and underline that its use yields in improved resolution.

### 3.4 S22019F Enables Accurate Isolation of NK Cells Expressing *KIR2DS1* Transcripts

**FIGURE 4:**
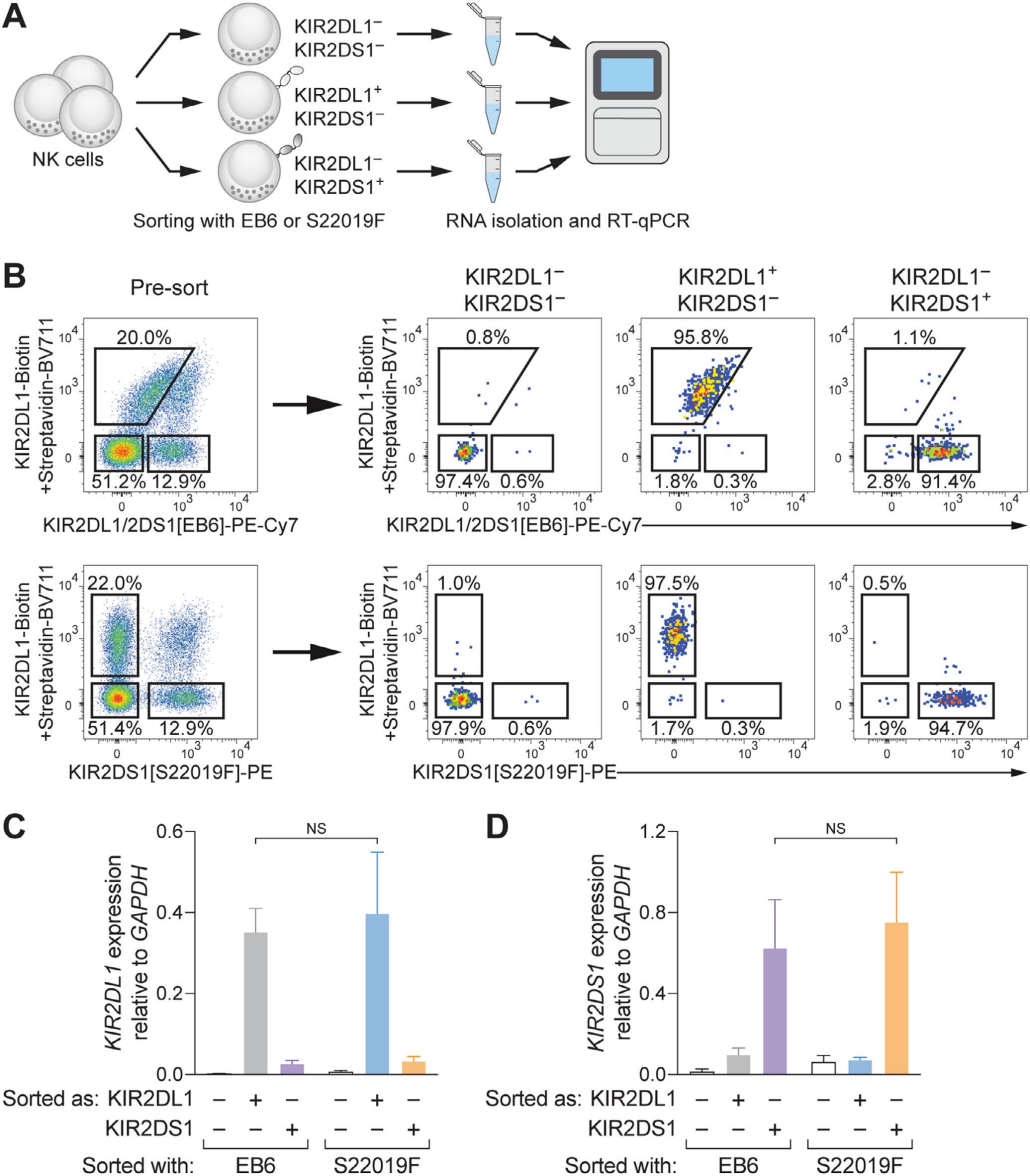
S22019F enables accurate isolation of NK cells expressing KIR2DS1 transcripts. (A) Schematic overview of sorting for subsequent RNA isolation and RT‐qPCR. (**B**) Representative sorting of viable CD14^−^ CD19^−^ CD3^−^ CD56^dim^ NK cells into KIR2DS1^−^KIR2DL1^−^, KIR2DL1^+^KIR2DS1^−^, and KIR2DL1^−^KIR2DS1^+^ subsets using EB6 (upper row) or S22019F (lower row) in combination with REA284. (C) Summary of *KIR2DL1* transcript abundance relative to GAPDH (*n* = 3 donors, bars indicate mean and error bars SEM; paired *t*‐test). (D) Summary of KIR2DS1 transcript abundance relative to *GAPDH* (*n* = 3 donors, bars indicate mean and error bars SEM; paired *t*‐test). NS: Not significant.

To further ascertain the specificity of S22019F and simultaneously explore its utility, we sorted primary NK cell subsets for subsequent RNA isolation and quantification of *KIR* transcripts by RT‐qPCR (Figure 4A). For this, we sorted KIR2DL1^−^KIR2DS1^−^, KIR2DL1^+^KIR2DS1^−^, and KIR2DL1^−^KIR2DS1^+^ CD56^dim^ NK cells, using either EB6 or S22019F in combination with REA284 (Figure 4B). Quantification of *KIR2DL1* transcripts uncovered high expression in KIR2DL1^+^KIR2DS1^−^ NK cells, independent of whether EB6 or S22019F was used for sorting (Figure 4C). Correspondingly, KIR2DL1^−^KIR2DS1^+^ NK cells displayed abundant expression of *KIR2DS1* transcripts, and the levels between cells sorted with EB6 or S22019F were equivalent (Figure 4D).

These findings confirm that S22019F specifically detects NK cells expressing *KIR2DS1* transcripts and highlight that S22019F enables the accurate isolation of KIR2DS1^+^ NK cells for downstream analyses.

### 3.5 S22019F Detects KIR2DS1 ^+^ NK Cells Upon Activation by HLA‐C*15

**FIGURE 5:**
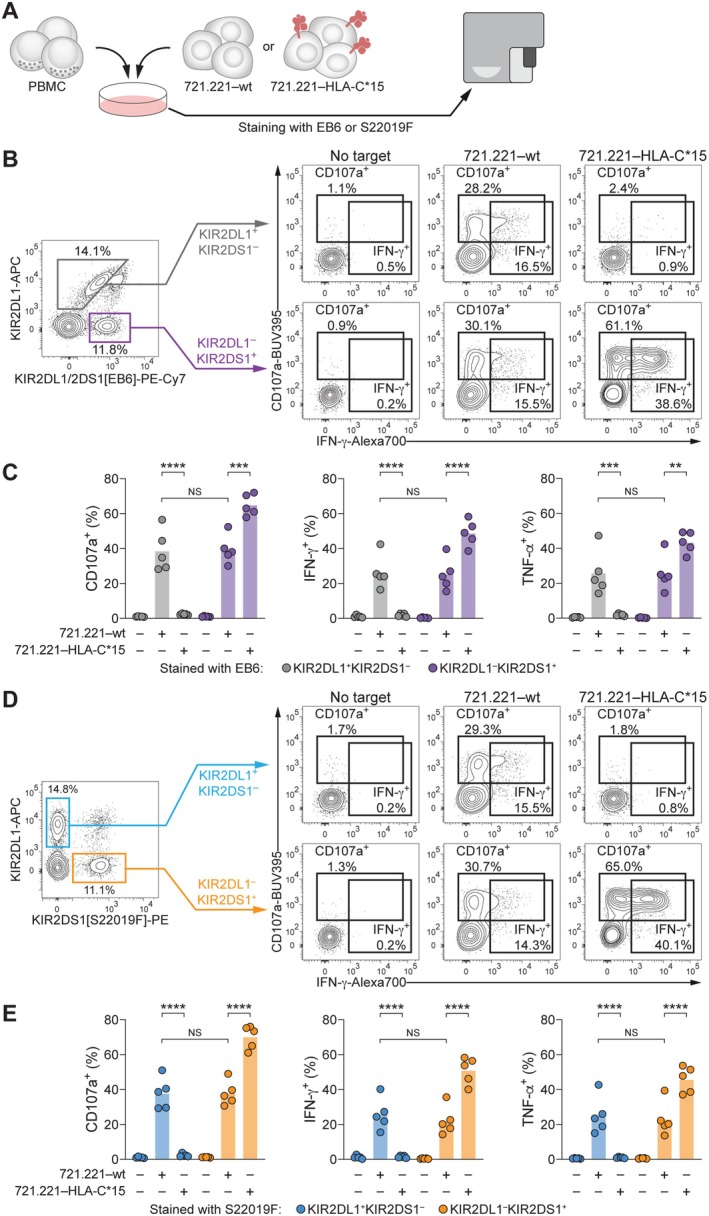
S22019 detects KIR2DS1^+^ NK cells upon activation by HLA‐C*15. (A) Schematic overview of PBMC co‐culture with target cells and subsequent flow cytometric analysis of NK cells. (B) Representative gating of KIR2DL1^+^KIR2DS1^−^ and KIR2DL1^−^KIR2DS1^+^ populations within viable CD14^−^CD19^−^CD3^−^CD56^dim^ NK cells using EB6 in combination with REA284 (left) and degranulation as well as IFN‐γ expression in the indicated conditions (right). (C) Summary of functional responses of KIR2DL1^+^KIR2DS1^−^ and KIR2DL1^−^KIR2DS1^+^ NK cells in the indicated conditions (*n* = 5 donors, bars indicate mean and each dot represents an individual donor; repeated‐measures one‐way ANOVA with Šídák multiple comparison test). (D) Representative gating of KIR2DL1^+^KIR2DS1^−^ and KIR2DL1^−^KIR2DS1^+^ populations within viable CD14^−^CD19^−^CD3^−^CD56^dim^ NK cells using S22019F in combination with REA284 (left) and degranulation as well as IFN‐γ expression in the indicated conditions (right). (E) Summary of functional responses of KIR2DL1^+^KIR2DS1^−^ and KIR2DL1^−^KIR2DS1^+^ NK cells in the indicated conditions (*n* = 5 donors, bars indicate mean and each dot represents an individual donor; repeated‐measures one‐way ANOVA with Šídák multiple comparison test). NS: Not significant, **p < 0.01, ***p <  0.001, and ****p < 0.0001.

Since KIR2DS1 is a functional activating receptor, we next assessed whether the binding of S22019F to KIR2DS1 is preserved upon engagement of its ligand HLA‐C. To this end, we co‐cultured PBMC with the HLA‐negative B cell lymphoblastoid cell line 721.221 (721.221–wt) or with transfectants expressing HLA‐C*15 (721.221–HLA‐C*15; Figure 5A; Figure S2A,B). Directly probing functional responses of KIR2DL1^+^KIR2DS1^−^ and KIR2DL1^−^KIR2DS1^+^ NK cells using EB6 in combination with REA284, we observed that KIR2DL1^+^KIR2DS1^−^ NK cells robustly degranulated and expressed IFN‐γ as well as TNF‐α upon co‐culture with 721.221–wt target cells, whereas their activity was fully inhibited by HLA‐C*15‐expressing targets. Conversely, KIR2DL1^−^KIR2DS1^+^ NK cells responded more strongly to 721.221–HLA‐C*15 than to 721.221–wt cells, consistent with the activating function of KIR2DS1 (Figure 5B,C; Figure S2C). Replacing EB6 with S22019F streamlined the identification of KIR2DL1^+^KIR2DS1^−^ and KIR2DL1^−^KIR2DS1^+^ populations and produced equivalent functional read‐outs (Figure 5D,E; Figure S2D).

These data demonstrate that S22019F faithfully detects KIR2DS1^+^ NK cells following HLA‐C‐mediated activation, indicating its suitability for functional investigations.

### 3.6 S22019F Does Not Cross‐React With KIR2DL3*005

**FIGURE 6:**
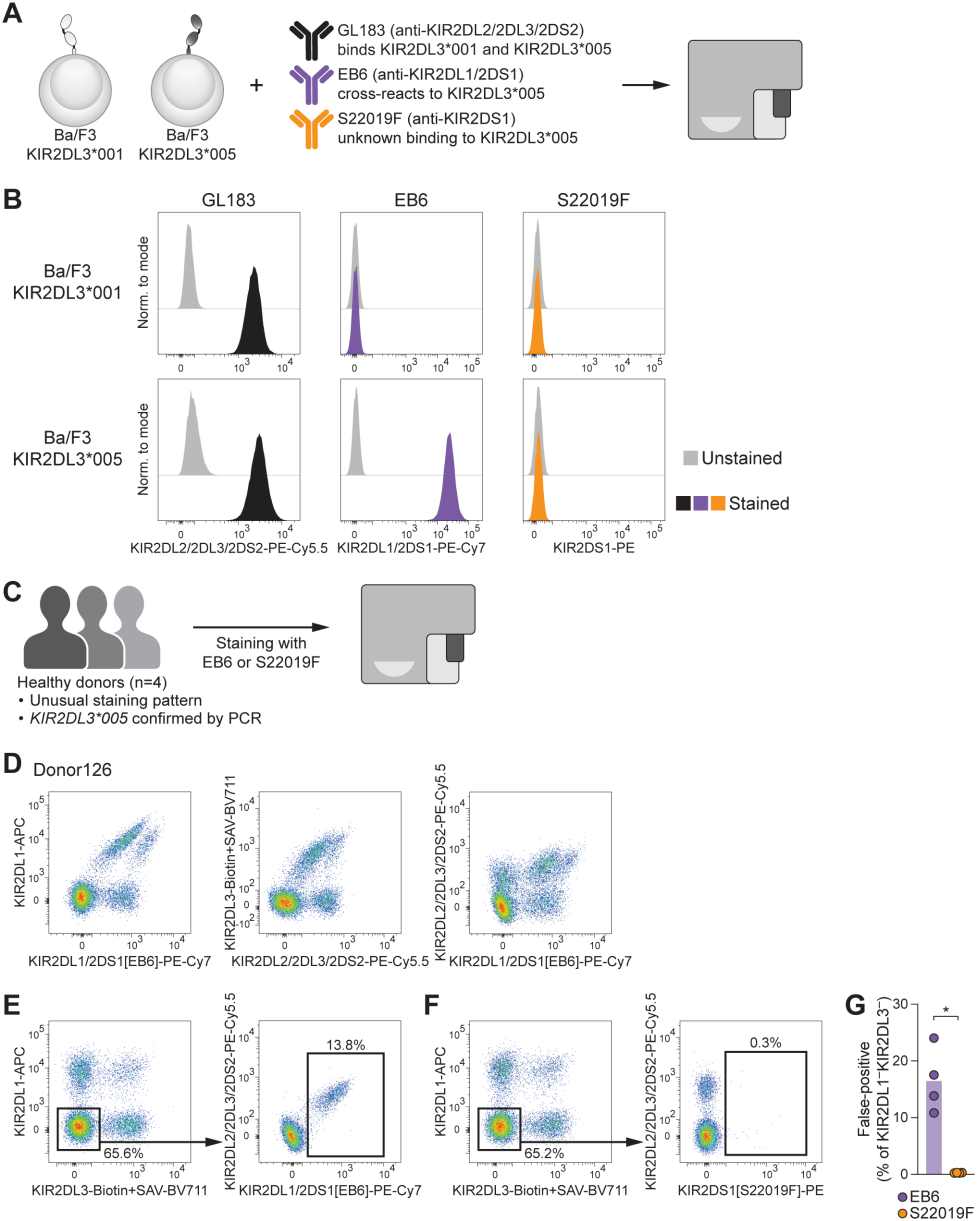
S22019F does not cross‐react with KIR2DL3*005. (A) Schematic overview of antibody binding assay. (**B**) Binding of anti‐KIR2DL2/2DS2/2DL3 GL183, anti‐KIR2DL1/2DS1 EB6 and S22019F to KIR2DL3*001‐transduced and KIR2DL3*005‐transduced Ba/F3 cells. One representative of two experiments is displayed. (C) Schematic overview of flow cytometric analysis of PBMC from healthy donors with confirmed presence of KIR2DL3*005. (D) Representative unusual staining pattern between anti‐KIR2DL2/2DS2/2DL3 GL183 and anti‐KIR2DL1/2DS1 EB6. (E) Representative false‐positive staining with EB6 due to cross‐reactivity to KIR2DL3*005. (F) Representative staining with S22019F. (G) Summary of false‐positive stainings (*n* = 4 donors, bars indicate mean and each dot represents an individual donor; paired *t*‐test). *p < 0.05. SAV, Streptavidin.

In addition to recognising both KIR2DL1 and KIR2DS1, EB6 is documented to cross‐react with KIR2DL3*005 allotypes [17]. Therefore, we investigated whether S22019F exhibits a similar cross‐reactivity by assessing antibody binding to Ba/F3 cells transduced with KIR2DL3*001 or KIR2DL3*005 (Figure 6A). As expected, the anti‐KIR2DL2/2DL3/2DS2 antibody GL183 labelled both cell lines and EB6 showed the described cross‐reactivity to KIR2DL3*005‐transduced Ba/F3 cells (Figure 6B). On the contrary, S22019F did not bind to either cell line (Figure 6B).

We next screened healthy donors for unusual staining patterns between EB6 and GL183, which are indicative of KIR2DL3*005 allotypes [14, 15], and confirmed the presence of KIR2DL3*005 by PCR (Figure S3A). Comparison of EB6 and S22019F binding profiles revealed that EB6 produced false‐positive populations in all four KIR2DL3*005 carriers, while S22019F did not (Figure 6C–G; Figure S3B–J).

We also assessed potential cross‐reactive binding to KIR2DS5 and found that S22019F did not bind to KIR2DS5*002‐transduced Ba/F3 cells (Figure S4A,B).

Taken together, these data confirm that S22019F does not cross‐react with KIR2DL3*005 or KIR2DS5.

### 3.7 S22019F Enables Detection and Analysis of KIR2DS1 ^+^ T Cells

**FIGURE 7:**
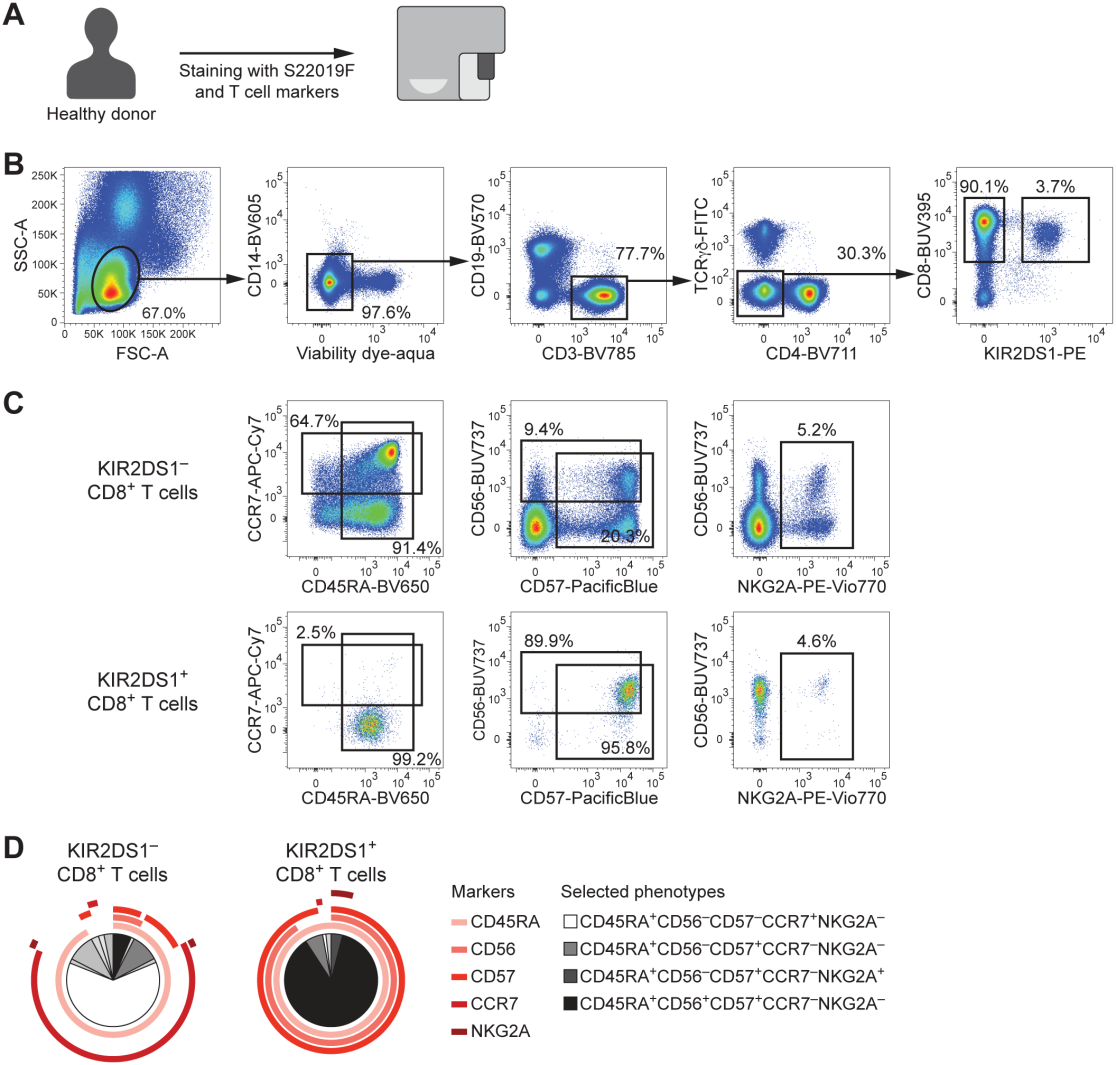
S22019F enables detection and analysis of KIR2DS1^+^ T cells. (A) Schematic overview of flow cytometric analysis of PBMC from a healthy donor carrying at least one B haplotype using S22019F and T cell markers. (B) Identification of KIR2DS1^+^ cells within viable CD14^−^ CD19^−^ CD3^+^ TCRγδ^−^ CD4^−^ CD8^+^ lymphocytes. (C) Assessment of CCR7, CD45RA, CD56, CD57, and NKG2A expression in KIR2DS1^−^ (upper row) and KIR2DS1^+^ (lower row) CD8^+^ T cells. (D) Combinatorial co‐expression profiling in KIR2DS1^−^ (left pie chart) and KIR2DS1^+^ (right pie chart) CD8^+^ T cells. Arcs indicate marker expression and slices represent co‐expression phenotypes.

Although NK cells are the primary KIR‐expressing immune cell population, KIR can also be found on other lymphocytes such as T cells [18]. Accordingly, we examined the potential applicability of S22019F in studying KIR^+^ T cells by incorporating S22019F into a panel of T cell markers (Figure 7A). With this approach, we readily detected a KIR2DS1^+^ population within viable CD14^−^ CD19^−^ CD3^+^ TCRγδ^−^ CD4^−^ CD8^+^ T lymphocytes (Figure 7B). Closer phenotypic inspection of KIR2DS1^+^ T cells uncovered dominant expression of CD45RA in the absence of CCR7 and uniform positivity for CD56 as well as CD57 (Figure 7C). Interestingly, the inhibitory receptor NKG2A was not enriched on KIR2DS1^+^ T cells, implying that independent mechanisms underlie the acquisition of these two NK cell receptors by T cells. Combinatorial co‐expression profiling using SPICE [12] underscored that KIR2DS1^−^ CD8^+^ T cells were primarily composed of CD45RA^+^CD56^−^CD57^−^CCR7^+^NKG2A^−^ naïve cells (Figure 7D). Conversely, KIR2DS1^+^ CD8^+^ T cells were predominantly characterised by a CD45RA^+^CD56^+^CD57^+^CCR7^−^NKG2A^−^ terminally differentiated phenotype (Figure 7D).

Collectively, these findings illustrate the value of S22019F for the detection and analysis of KIR2DS1^+^ T cells.

## 4 Discussion

The activity of NK cells is controlled by a variety of combinatorially expressed activating and inhibitory receptors, coupling the receptor repertoire directly to NK cell function. Genetic variation in the KIR family and their HLA ligands is a major driver of diversity within the NK cell compartment [19]. Consequently, reagents that accurately detect the expression of single KIR proteins are critical for advancing fundamental as well as clinical studies of NK cells.

Here, we demonstrate that the newly generated antibody S22019F selectively recognises KIR2DS1. Due to this unique specificity, S22019F outperforms the current state‐of‐the‐art dual‐specific anti‐KIR2DL1/KIR2DS1 antibody EB6, which requires combination with a KIR2DL1‐specific reagent [8]. In addition, combining S22019F with an anti‐KIR2DL1 antibody enables clear and unambiguous identification of KIR2DS1^−^KIR2DL1^+^, KIR2DS1^+^KIR2DL1^−^, and KIR2DS1^+^KIR2DL1^+^ subsets, in contrast to the difficulty of separating these populations when using EB6 with anti‐KIR2DL1. Another advantage of S22019F is that its use precludes false‐positive results compared to the documented cross‐reactivity of EB6 to KIR2DL3*005 allotypes [17]. Moreover, S22019F allows to directly label the entire KIR2DS1^+^ population in one staining step, contrary to EB6 requiring a secondary staining step to mark all KIR2DS1^+^ NK cells [11], likely caused by steric hinderance with anti‐KIR2DL1 antibodies. Besides sequence homology between KIR, allelic diversity within KIR2DS1 may alter the binding of antibody reagents, similar to what has been observed for KIR2DL1 [20]. Although the binding profile of S22019F matched the staining by EB6 in eight A/A and eight B/x donors, future studies are required to unequivocally exclude that certain allelic variants of KIR2DS1 may not be recognised by this reagent. In summary, SS2019F shows several key advantages compared to currently used reagents and, as such, will facilitate and improve the analysis of KIR2DS1^+^ NK cells and T cells.

Activating KIR play a central role in the graft‐versus‐leukaemia effect of NK cells following allogeneic haematopoietic stem cell transplantation. Patients receiving grafts from KIR genotype B/x donors exhibit superior survival compared to those receiving A/A grafts [21]. Presence of the *KIR2DS1* gene in the graft specifically confers protection against relapse [5], consistent with the activation of KIR2DS1^+^ NK cell clones by group C2 HLA‐C molecules on allogeneic leukaemic blasts [22]. Moreover, KIR2DS1^+^ NK cell clones efficiently eliminate dendritic cells and activated T cells in vitro, potentially limiting graft‐versus‐host disease and improving engraftment [23]. Despite these benefits, current donor selection is based solely on genotype, without considering actual protein expression patterns or functional capacities. Therefore, directly measuring the frequency of KIR2DS1^+^ NK cells and assessing their functional activity using S22019F could represent a powerful predictive tool to further improve the selection of optimal donors.

Beyond instructing the killing of leukaemic cells, KIR2DS1 is also implicated in HLA‐C‐dependent recognition of cytomegalovirus (CMV) in vitro [6]. This notion is supported by protective effects of *KIR2DS1*, *KIR2DS3*, and *KIR3DS1* genes against CMV reactivation [24] and by the expansion of NK cell sub‐populations bearing activating KIR including KIR2DS1 in response to CMV infection [25, 26], although the exact mechanisms remain enigmatic. Whether CMV‐derived peptides presented on HLA‐C mediate recognition by KIR2DS1, analogous to KIR2DS2 and hepatitis C virus [7], or whether CMV encodes a protein ligand for KIR2DS1 as reported for *Plasmodium falciparum* [27], remains unknown. Hence, S22019F will likely expedite future studies of KIR2DS1‐mediated NK cell responses against CMV.

Presence of the *KIR2DS1* gene is not purely beneficial, as it is also associated with psoriasis [28, 29] and multiple sclerosis [30]. Using S22019F in this context will enable pinpointing the cellular mediators that underlie the genetic associations. Since KIR2DS1 is expressed by subsets of NK cells and T cells, an essential first step will be to determine whether KIR2DS1‐bearing T cells drive these autoimmune conditions, or whether KIR2DS1^+^ NK cells contribute to dysregulation. This approach will be crucial for better understanding the pathogenesis of these autoimmune disorders.

In summary, our in‐depth characterisation demonstrates that the antibody S22019F specifically recognises KIR2DS1. S22019F thus enables accurate determination of the frequency, phenotype, and functional features of KIR2DS1‐bearing lymphocytes in health and disease.

## Author Contributions

Conceptualization: Q.H. Methodology: C.B., M.L., T.O. Investigation: E.B., Ju.Me., J.W., Q.H. Resources: Ja.Mi., H.‐G.L., P.B., C.W. Funding acquisition: P.B., Q.H. Supervision: E.B., Q.H. Writing, original draft: Ja.Mi., H.‐G.L., Q.H. Writing, review and editing: All authors.

## Funding

This work was supported by Precision Health in Schleswig‐Holstein. Schleswig‐Holstein Excellence Chair Program; Deutsche Forschungsgemeinschaft (under Germany’s Excellence Strategy), EXC 2167/2 ‐ 390884018; Vetenskapsrådet, 2024‐02358; Åke Wiberg Stiftelse, M24‐0004; Jeanssons Stiftelser, J2024‐0002; KI Foundations, 2024‐02467; Magnus Bergvalls Stiftelse, 2024‐1259; and the Jonas Soderquist Foundation.

## Conflicts of Interest

M.L., T.O., and C.W. are employees of BioLegend Inc. H.‐G.L. and Q.H. are consultants for Vycellix Inc., unrelated to this work. The other authors declare no conflicts of interest.

## Supporting information


**Figure S1:** Unbiased assessment of S22019F specificity. Related to Figure 3.
**Figure S2:** S22019F detects KIR2DS1^+^ NK cells upon activation by HLA‐C*15. Related to Figure 5.
**Figure S3:** S22019F does not cross‐react with KIR2DL3*005. Related to Figure 6.
**Figure S4:** S22019F does not cross‐react with KIR2DS5. Related to Figure 6.
**Table S1:** Antibodies used in this study.

## Data Availability

The data that support the findings of this study are available from the corresponding author upon reasonable request.
