# Supplementary material for: Antibody S22019F Selectively Recognises KIR2DS1 and Enables Analysis of KIR2DS1 + NK Cells and T Cells
Source: HLA. 2026 May 13;107:e70748. doi: 10.1111/tan.70748 (PMC13169263; doi:10.1111/tan.70748)
Supplement: Supplementary file 1 — Figure S1: Unbiased assessment of S22019F specificity. Related to Figure 3. Figure S2: S22019F detects KIR2DS1+ NK cells upon activation by HLA‐C*15. Related to Figure 5. Figure S3: S22019F does not cross‐react with KIR2DL3*005. Related to Figure 6. Figure S4: S22019F does not cross‐react with KIR2DS5. Related to Figure 6. Table S1: Antibodies used in this study. [file TAN-107-e70748-s001.docx]

**Antibody S22019F Selectively Recognizes KIR2DS1 and Enables Analysis of KIR2DS1⁺ NK Cells and T Cells**

Eleni Bilev^1^, Julia Meinecke^1^, Jascha Wienberg^2^, Caroline Boulouis^1^, Michael Li^3^, Takatoku Oida^3^, Jakob Michaëlsson^1^, Hans-Gustaf Ljunggren^1^, Petra Bacher^2^, Carsten Wiethe^3^, Quirin Hammer^1,2^.

^1^ Center for Infectious Medicine, Department of Medicine Huddinge, Karolinska Institutet, Karolinska University Hospital Huddinge, Stockholm, Sweden.

^2^ Institute of Medical Immunology, Christian-Albrechts-University of Kiel and University Medical Center Schleswig-Holstein, Kiel, Germany.

^3^ BioLegend Inc., San Diego, California, USA.

Correspondence: Quirin Hammer ([quirin.hammer@uksh.de](mailto:quirin.hammer@uksh.de))

**Supplementary Figures**

| 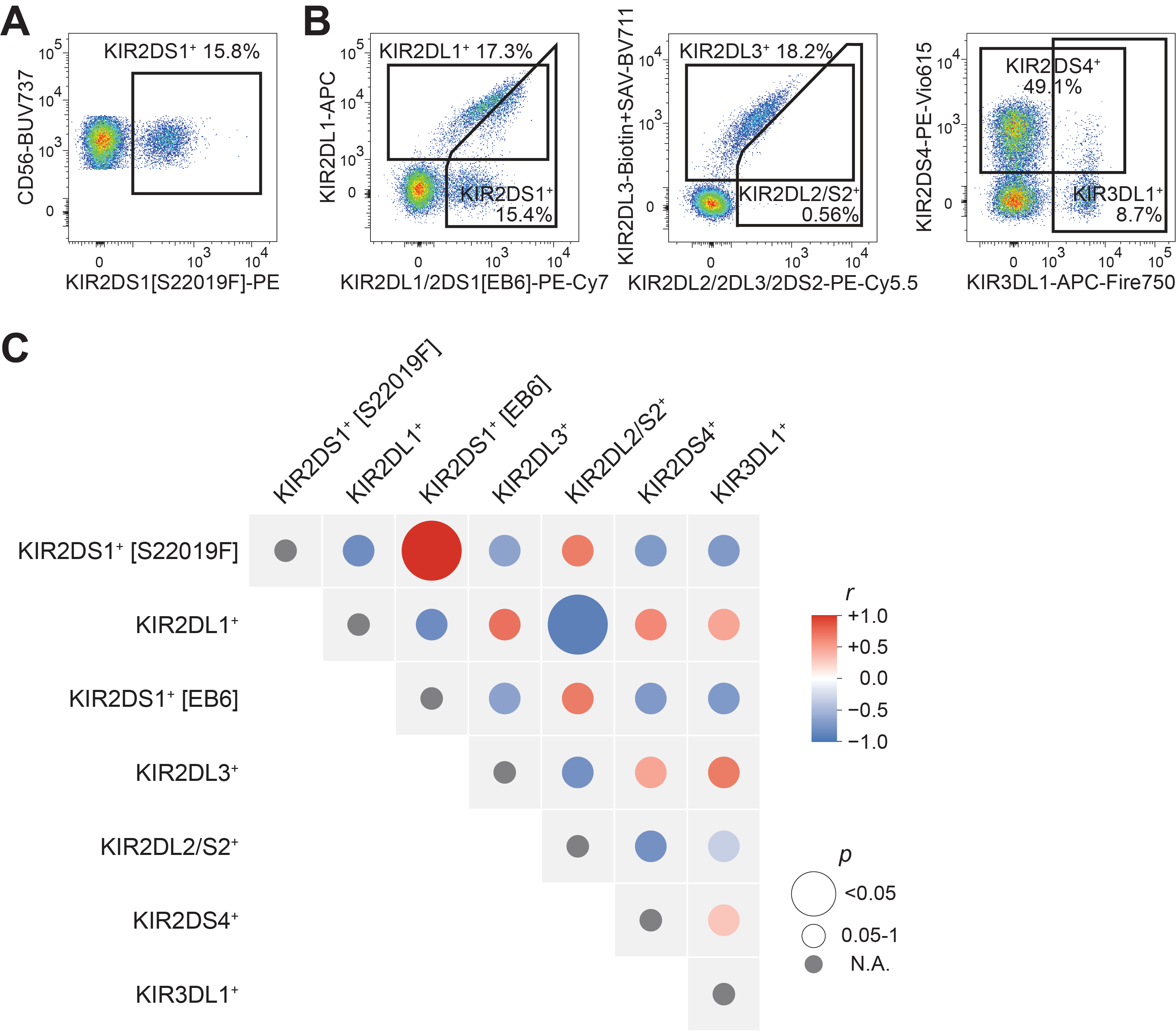 |
| --- |
| **Supplementary Figure S1. Unbiased assessment of S22019F specificity. Related to Figure 3.**  PBMC from six KIR B/x genotype donors were stained with (**A**) S22019F or (**B**) a panel covering KIR2DL1 (REA284), KIR2DL1/2DS1 (EB6), KIR2DL3 (REA147), KIR2DL2/2DL3/2DS2 (GL183), KIR3DL1 (DX9), and KIR2DS4 (REA860). (**C**) Correlation matrix displaying pairwise correlations computed between KIR^+^ populations (n=6 donors; bubble color indicates Pearson correlation coefficient *r* and bubble size represents *p* value; N.A., not available; SAV, Streptavidin.) |

| 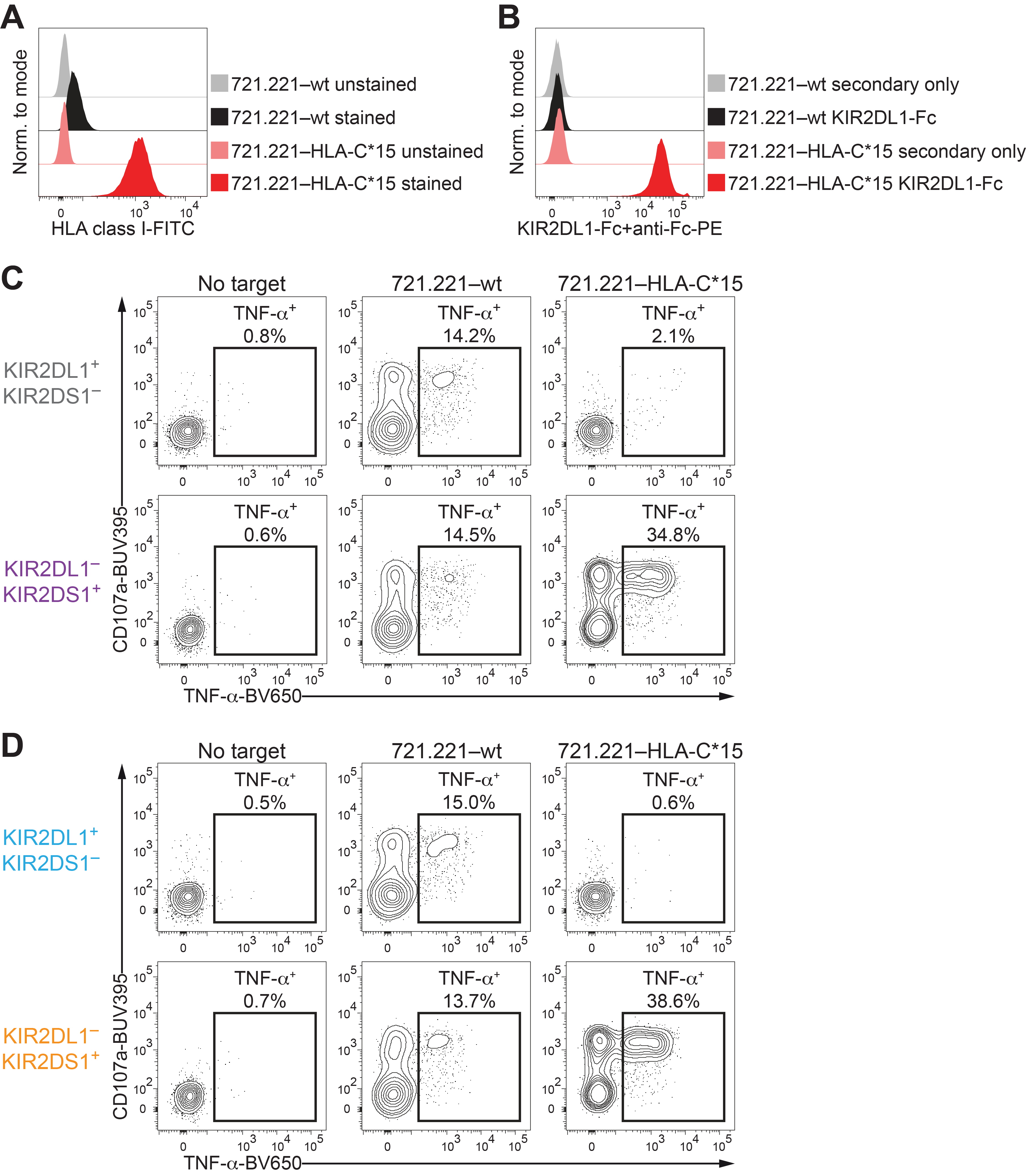 |
| --- |
| **Supplementary Figure S2. S22019F detects KIR2DS1^+^ NK cells upon activation by HLA-C*15. Related to Figure 5.**  (**A**) HLA class I expression on 721.221–wt and 721.221–HLA-C*15 cells. (**B**) Binding of recombinant KIR2DL1-Fc chimera molecules to 721.221–wt and 721.221–HLA-C*15 cells as detected with goat anti-human IgG-Fc-PE secondary antibody. (**C**) Representative TNF-α expression of KIR2DL1^+^KIR2DS1^–^ and KIR2DL1^–^KIR2DS1^+^ NK cells using EB6 in combination with REA284. (**D**) Representative TNF-α expression of KIR2DL1^+^KIR2DS1^–^ and KIR2DL1^–^KIR2DS1^+^ NK cells using S22019F in combination with REA284. |

| 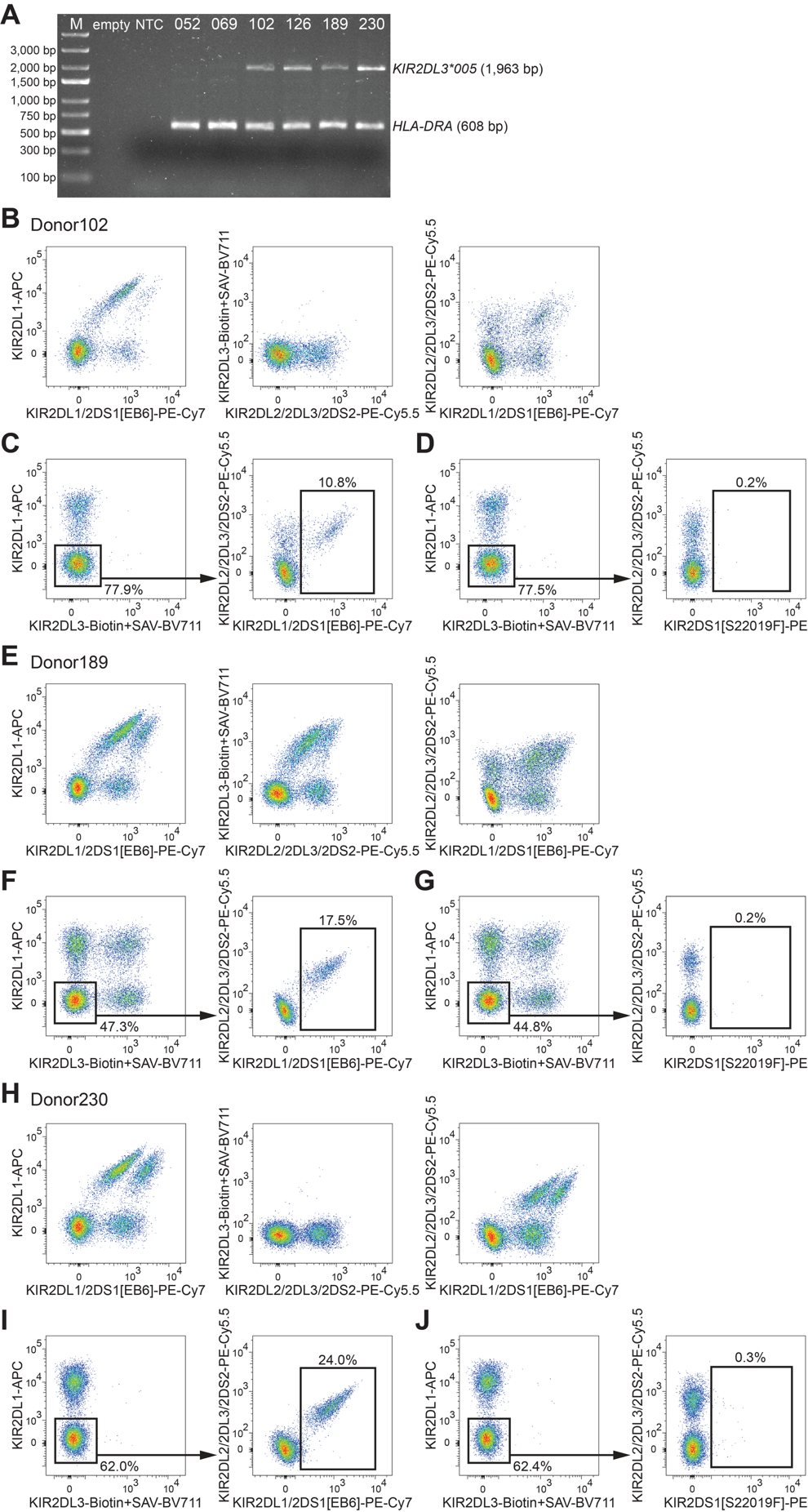 |
| --- |
| **Supplementary Figure S3. S22019F does not cross-react with KIR2DL3*005. Related to Figure 6.** *(legend on next page)*  **Supplementary Figure S3. S22019F does not cross-react with KIR2DL3*005. Related to Figure 6.**  (**A**) Detection of *KIR2DL3*005* by PCR in DNA from candidate donors. M: molecular weight marker. NTC: no-template-control. Two donors without *KIR2DL3*005* and four donors with *KIR2DL3*005* are displayed. (**B-J**) Stainings in three donors carrying *KIR2DL3*005*, including unusual staining pattern between anti-KIR2DL2/2DS2/2DL3 GL183 and anti-KIR2DL1/2DS1 EB6 (B, E, H), false-positive stainings with EB6 due to cross-reactivity to KIR2DL3*005 (C, F, I), and stainings with S22019F (D, G, J). SAV, Streptavidin. |

| 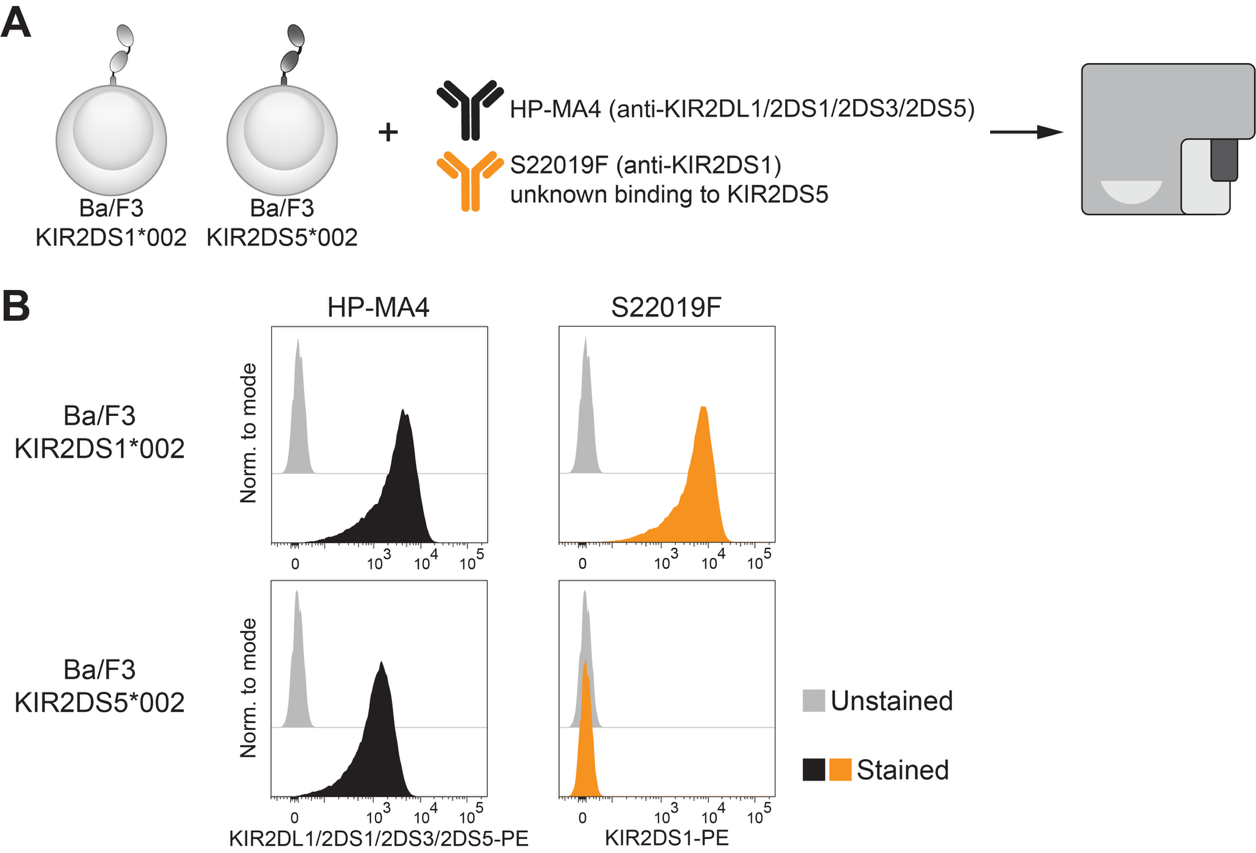 |
| --- |
| **Supplementary Figure S4. S22019F does not cross-react with KIR2DS5. Related to Figure 6.**  (**A**) Schematic overview of antibody binding assay. (**B**) Binding of anti-KIR2DL1/2DS1/2DS3/2DS5 HP-MA4 and S22019F to KIR2DS1*002-transduced and KIR2DS5*002-transduced Ba/F3 cells. One representative of two experiments is displayed. |

**Supplementary Table S1. Antibodies used in this study.**

| Reagent (clone) | Manufacturer | Cat# | RRID |
| --- | --- | --- | --- |
| Anti-CCR7-APC-Cy7 (G043H7) | BioLegend | 353212 | AB_10916390 |
| Anti-CD107a-BUV395 (H4A3) | BD Biosciences | 565113 | AB_2739073 |
| Anti-CD14-BV605 (M5E2) | BioLegend | 301834 | AB_2563798 |
| Anti-CD16-BV785 (3G8) | BioLegend | 302046 | AB_2563803 |
| Anti-CD19-BV570 (HIB19) | BioLegend | 302236 | AB_2563606 |
| Anti-CD3-BV785 (UCHT1) | BioLegend | 300472 | AB_2687178 |
| Anti-CD3-PE-Cy5 (UCHT1) | BioLegend | 300410 | AB_314064 |
| Anti-CD4-BV711 (OKT4) | BioLegend | 317440 | AB_2562912 |
| Anti-CD45RA-BV650 (HI100) | BD Biosciences | 563963 | AB_2738514 |
| Anti-CD56-BUV737 (NCAM16.2) | BD Biosciences | 612767 | AB_2860005 |
| Anti-CD57-PacificBlue (HNK-1) | BioLegend | 359608 | AB_2562459 |
| Anti-CD8-BUV395 (RPA-T8) | BD Biosciences | 563795 | AB_2722501 |
| Anti-HLA class I-FITC (W6/32) | BioLegend | 311404 | AB_314873 |
| Anti-IFN-γ-Alexa700 (B27) | BD Biosciences | 557995 | AB_396977 |
| Anti-IgG Fc-PE (polyclonal) | ThermoFisher | 12-4998-82 | AB_465926 |
| Anti-panKIR2D-APC (NKVFS1) | Miltenyi | 130-132-195 | AB_3663862 |
| Anti-KIR2DL1-APC (REA284) | Miltenyi | 130-120-444 | AB_2752101 |
| Anti-KIR2DL1-Biotin (REA284) | Miltenyi | 130-120-445 | AB_2752102 |
| Anti-KIR2DL1/DS1-PE-Cy7 (EB6) | Beckman Coulter | A66899 | AB_2801261 |
| Anti-KIR2DL1/DS2/DS3/DS5-PE (HP-MA4) | BioLegend | 339506 | AB_2130374 |
| Anti-KIR2DL2/L3/S2-PE-Cy5.5 (GL183) | Beckman Coulter | A66900 | AB_2857331 |
| Anti-KIR2DS1-PE (S22019F) | BioLegend | 335008 | *Pending* |
| Anti-KIR2DS4-PE-Vio615 (REA860) | Miltenyi Biotec | 130-114-623 | AB_2655370 |
| Anti-KIR2DL3-Biotin (REA147) | Miltenyi Biotec | 130-100-127 | AB_2655344 |
| Anti-KIR3DL1-APC-Fire750 (DX9) | BioLegend | 312722 | AB_2687394 |
| Anti-NKG2A-PE-Vio770 (REA110) | Miltenyi Biotec | 130-113-567 | AB_2726172 |
| Anti-NKG2A-VioBrightFITC (REA110) | Miltenyi Biotec | 130-113-568 | AB_2726173 |
| Anti-TCRγδ-FITC (REA591) | Miltenyi Biotec | 130-113-511 | AB_2733697 |
| Anti-TNF-α-BV650 (MAb11) | BioLegend | 502938 | AB_2562741 |
| Streptavidin-BV711 | BD | 563262 | AB_2869478 |
